# Hypertension self-care practice and associated factors among patients in public health facilities of Dessie town, Ethiopia

**DOI:** 10.1186/s12913-019-3880-0

**Published:** 2019-01-21

**Authors:** Sewunet Ademe, Fekadu Aga, Debela Gela

**Affiliations:** 1 Department of Adult Health Nursing, School of Nursing & Midwifery, Wollo University, P.O. Box: 1145, Dessie, Ethiopia; 2 Department of Nursing, School of Nursing & Midwifery, College of Health Science, Addis Ababa University, P.O. Box: 9083, Addis Ababa, Ethiopia; 3 Department of Nursing, School of Nursing & Midwifery, College of Health Science, Addis Ababa University, P.O. Box: 4412, Addis Ababa, Ethiopia

**Keywords:** Self-care practice, Hypertension, Social support, Self-care agency, Ethiopia

## Abstract

**Background:**

Hypertension self-care practice is essential for blood pressure control and reduction of hypertension complications. Nevertheless, we know little concerning hypertension self-care practice in Ethiopia. The purpose of this study was to assess hypertension self-care practice and associated factors among patients in public health facilities in Dessie town, Ethiopia.

**Methods:**

In this cross-sectional study, 309 hypertensive patients (mean age 58.8 years, 53.4% women) completed the interviewer-administered questionnaire in Amharic language. Descriptive and logistic regression analyses were conducted using SPSS version 22.

**Result:**

The mean score for hypertension self-care was 37.7 ± 8.2 and 51% scored below the mean. Divorced participants (AOR = 0.115, 95% CI = 0.026, 0.508, *p*-value < 0.01) and those who lack source of information (AOR = 0.084, 95% CI = 0.022, 0.322, *p*-value < 0.01) were less likely to have good self-care practice. But, participants who had convenient place for exercise (AOR = 2.968, 95% CI = 1.826, 4.825, *p*-value < 0.01), who had good social support (AOR = 2.204, 95% CI = 1.272, 3.821, *p*-value < 0.01), who had traditional clergy-based teaching (AOR = 2.209, 95% CI = 1.064, 4.584, *p*-value < 0.05), and who had good self-care agency (AOR = 1.222, 2.956, *p*-value < 0.05) were more likely to have good self-care practice.

**Conclusion:**

Most of the study participants reported poor self-care practices. Factors associated with hypertension self-care practice are marital status, education, source of self-care information, place for exercise, social support, and self-care agency. Targeted interventions are needed to improve hypertension self-care practice.

## Background

Hypertension or high blood pressure is the leading global health problem which is prevalent in all regions and countries of the world [1–4]. It is one of the three leading risk factors for global disease burden accounting for 7% of the global disability-adjusted life years (DALYs) [4]. About 31.1% of the world adult population lives with hypertension, and 28.5% are in high-income countries while 31.5% are in low-and middle-income countries [5]. The age-standardized prevalence of hypertension is 25.9% among different population groups in the sub-Saharan Africa [6]. In Ethiopia, the prevalence of hypertension is 31.5% among men and 28.9% among women in Addis Ababa, [7] 18.8% in Sidama Zone, [8] 28.3% in Gondar, [9] 13.2% in Jimma, [10] and 11% in Mekele [11]. Thus, hypertension has become an important health threat in this resource limited country – Ethiopia.

Hypertension is an important risk factor for cardiovascular disease and mortality. According to the World Health Organization’s (WHO) report, complications of hypertension accounts for 9.4 million of the annual 17 million worldwide deaths from cardiovascular disease [12]. The report further explains that hypertension is responsible for approximately 45% of deaths resulting from heart disease and 51% of deaths from stroke. In addition, hypertension is a risk factor for renal and eye diseases [13, 14].

Studies have demonstrated that self-care practice is essential for blood pressure control and reduction of hypertension complications of cardiovascular and renal diseases [15–17]. Hypertension self-care has been defined as “a dynamic and active process requiring knowledge, attitude, discipline, determination, commitment, self-regulation, empowerment and self-efficacy” [18]. It involves medication taking, consumption of low-sodium and low-fat diet, exercise, limiting alcohol drinking, not smoking, weight reduction, self-monitoring blood pressure, regular healthcare visit, and reducing stress [19]. The use of a combination of diet rich in fruits, vegetables, and low-fat dairy products with reduced saturated and total fat lowers systolic blood pressure by 5.5 mmHg and diastolic blood pressure by 3 mmHg [20–22]. Restricting sodium intake to no more than 2.4 g per day would lowers systolic blood pressure by 2–8 mmHg in hypertensive patients [23–25]. Performing regular aerobic physical activity at least 30 min daily and 150 min per week would reduces systolic blood pressure by 4–9 mmHg [26, 27]. A 50% reduction of daily alcohol consumption would also lower systolic blood pressure by 2–7 mmHg and diastolic blood pressure by 2–5 mmHg [28, 29]. Reducing weight to maintain normal body mass index (18.5–24.9 Kg/m^2^) would lower systolic blood pressure by 3–7 mmHg and diastolic blood pressure by 3–9 mmHg [30–32].

Nevertheless, hypertensive patients often do not implement the recommended self-care practices and ultimately suffer from uncontrolled blood pressure. According to a recent study, about 50% of hypertensive patients in southwest Ethiopia live with uncontrolled blood pressure [33]. A multitude of factors may affect hypertension self-care practice. These may include demographic factors such as age, education, employment, and health literacy [34–37], illness duration [34, 36], empowerment factors such as self-care agency and self-efficacy [34, 38, 39], hypertension knowledge [40], and social support [38, 41]. However, we know little concerning hypertension self-care in Ethiopia. Therefore, the purpose of this study was to assess hypertension self-care practice and associated factors among patients in public health facilities of Dessie town, Ethiopia. This study was informed by the Health-Promoting Self-Care System Model (HPSCSM) which is used as a framework for identifying and explaining patterns among factors that influence the decision-making, performance and outcomes of health-promoting lifestyles [42]. The HPSCSM was built by synthesizing concepts from Orem’s Self-Care Deficit Nursing Theory [43], Cox’s Interaction Model of Client Health Behavior [44], and Pender’s Health Promotion Model [45]. The HPSCSM proposes that demographics, social factors, environmental factors, perceived health state, and health care expenditure influence individual’s performance of health-promoting self-care. The association between these variables and hypertension self-care practice was analyzed in this study.

## Methods

### Design and sample

This was institution-based cross-sectional study conducted in 4 public health institutions in Dessie town using proportionally allocated stratified sampling method. The study conformed to the fundamental principles of research ethics and approved by the Institutional Review Board (IRB) of the College of Health Science at Addis Ababa University. Permission to conduct the research was obtained from the authorities in the study settings and informed consents were secured from each participant. A total of 309 participants, with 98% of response rate, consented and recruited from one public hospital and three health centers in Dessie town from March to May 2017. The sample size was determined using a single population proportion formula based on the following assumptions: considering 95% of confidence level, 5% margin of error, and 50% population proportion. A sample size correction formula was also applied since the total hypertensive patient population was less than 10,000. The study participants were finally recruited using systematic sampling technique with the patients’ follow up registry serving as a sampling frame in each participating institution. Every 4 patient in the registry was recruited from each institution with the first one determined using a lottery method. Patients were included in this study if they meet the following criteria: being on hypertension follow up at least for the last 6 months; 18 years or older; and can read and write in Amharic language. Patients were excluded if they were critically sick and have serious mental or cognitive impairment. The participants completed the interviewer-administered questionnaire immediately after giving consent.

### Measures

Sociodemographic information such as age, sex, ethnicity, income, marital status, residence place, income, and educational status were collected using eight structured items included in the interviewer-administered questionnaire. A single dichotomous item was used to measure family history of hypertension. Duration since diagnosed as hypertensive and source of information about hypertension self-care were respectively measured using single multiple-choice type items. We have also used one dichotomous item to measure availability of convenient place to perform physical activity.

The behavioral scale of ***Hypertension Self-Care Profile (HBP-SCP)*** [46] was used to measure the self-care practice of participants in this study. The HBP-SCP is a 20-item measure with each question having 4 response options: not at all = 1, sometimes = 2, often = 3, and always = 4. A total score can range from 20 to 80 with higher score indicating better self-care practice. The behavioral scale of the original HBP-SCP [46] had Cronbach’s alpha of 0.83 where as alpha of approximately 0.85 was recently reported by two studies from Singapore [47, 48].

The ***Appraisal of Self-Care Agency – Revised (ASAS-R)*** [49, 50] was used to measure self-care agency in this study. The ASAS-R is a 15-item measure that assesses the extent of self-care agency of individuals on a 5-point Likert scale ranging from 1 = totally disagree to 5 = totally agree. The total score can range from 15 to 75 with higher score indicating better self-care agency. A Cronbach’s alpha of 0.89 was reported for the original version of ASAS-R [49].

The ***Multidimensional Scale of Perceived Social Support (MSPSS)*** was used to measure subjectively assessed social support [51, 52]. The MSPSS is a 12-item measure of the perceived adequacy of social support on a 7-point Likert-type scale ranging from 1 = very strongly disagree to 7 = very strongly agree. The total score range from 12 to 84 with higher score indicating better perceived social support. Cronbach’s alpha of 0.87 was reported for patient groups [53].

### Data analysis

Data was entered into the Statistical Package for Social Sciences (SPSS) version 22 and then checked and cleaned. Descriptive statistics were calculated to describe the characteristics of the participants and the measured variables. Mean with standard deviation and percentages were used to present the descriptive statistics. Pearson’s correlation was computed to explore the bivariate correlation between hypertension self-care, self-care agency, and social support scores. In preparation for logistic regression analysis, we have dichotomized the HBP-SCP score as follow: good self-care practice for a score equal to or above the mean and poor self-care practice for a score below the mean. We have also used the same procedure to dichotomize the ASAA-R and MSPSS scores. The sociodemographic variables, source of information, place of exercise, social support, and self-care agency associations with self-care were analyzed first by using simple logistic (bivariate) regression model. Then, we used variable selection method for the multiple logistic analyses rather than pre-specifying the model because of lack of theoretical bases that serve as priori. Thus, only those variables with *p*-value less than or equal to 0.2 were taken as candidate for multiple logistic regression analysis. A p-value cut-off 0.2 was used to reduce the number of variables entered in the regression model presuming that there would not be much change for the variables with p-value more than 0.2. In both simple and multiple regression models, the statistical significance of associations between variables were determined using odds ratios with 95% confidence interval (CI) and *p*-values below 0.05.

## Result

### Participant characteristics

Table 1 shows that the participants mean age was 58.8, the majority were between 40 and 60 years (*n* = 142, 49.1%), female (*n* = 165, 53.4%), Muslims (*n* = 158, 51.1%), urban dwellers (*n* = 260, 84.1%), ethnic Amhara (*n* = 288, 93.8), and married (*n* = 214, 69.3%). From the total participants, 139 (45%) were farmers, 90 (29.1%) had family history of hypertension, 186 (60.2%) got information about self-care through health education, 180 (58.3%) lived with hypertension for more than 2 years, and 169 (54.7%) had no convenient place for physical activity. Table 1 also shows the disaggregated mean scores of the HBP-SCP.

**Table 1:** Characteristics of the study participants (*N* = 309)

Characteristics		n (%)	HBP-SCP Score, Mean (SD)
Age (in years): Mean = 58.8	<40 years	26 (8.4)	40.6 (7.8)
	40–60 years	142 (49.1)	37.4 (8.2)
	>60 years	130 (42.1)	37.6 (8.4)
Gender:	Male	144 (46.6)	39.2 (9.0)
	Female	165 (53.4)	36.4 (7.3)
Religious affiliation:	Orthodox	143 (46.3)	39.1 (8.6)
	Muslim	158 (51.1)	36.5 (7.8)
	Protestant	5 (1.6)	40.4 (8.7)
Residence place:	Urban	260 (84.1)	37.9 (8.4)
	Rural	49 (15.9)	36.8 (7.6)
Ethnicity:	Amhara	288 (93.8)	37.7 (8.2)
	Tigre	14 (4.3)	38.6 (8.9)
	Oromo	4 (1.3)	39.7 (9.0)
	Others	1 (0.6)	–
Marital status:	Single	20 (6.5)	40.4 (8.7)
	Married	214 (69.3)	38.1 (8.3)
	Divorced	28 (9.1)	32.8 (6.7)
	Widowed	47 (15.2)	37.1 (7.8)
Educational status:	Unable to read & write	100 (32.4)	34.4 (6.6)
	Traditional clergy-based teaching	59 (19.1)	37.2 (7.9)
	Primary school	69 (22.3)	38.2 (8.5)
	Secondary school	35 (11.3)	40.0 (8.4)
	College/University	46 (14.9)	44.4 (7.7)
Occupation:	Farmer	139 (45.0)	35.5 (6.4)
	Government employee	49 (15.9)	42.6 (8.1)
	Private employee	24 (7.8)	37.2 (7.8)
	Merchant	59 (19.1)	38.3 (8.8)
	Others	37 (12)	39.7 (11.0)
Monthly income (in ETB):	<500	84 (26.2)	37.4 (9.1)
	501–1000	84 (27.5)	35.1 (8.2)
	>1000	141 (46.3)	39.5 (7.3)
Family history of HBP:	Yes	90 (29.1)	38.0 (8.2)
	No	219 (70.9)	37.6 (8.3)
Source of information:	Books	28 (9.1)	45.4 (8.6)
	News	1 (0.3)	–
	Health education	186 (60.2)	38.8 (8.4)
	No information	94 (30.4)	33.4 (4.9)
Duration with HBP:	<6 months	1 (0.3)	–
	6months – 2 years	126 (40.8)	37.0 (7.2)
	>2 years	180 (58.3)	38.3 (8.9)
Place for exercise:	Yes	140 (45.3)	40.2 (9.2)
	No	169 (54.7)	36.1 (7.1)

*SD* Standard Deviation, *HBP-SCP* Hypertension Self-Care Profile, *HBP* Hypertension, *ETB* Ethiopian Currency

### Self-care practice and correlations

As presented in Table 2, the overall mean score for hypertension self-care practice was 37.7 ± 8.2. From the total participants, 51% scored below the mean on the HBP-SCP, indicating poor self-care practice. On the other hand, 52.8 and 70.2% scored equal to or above the mean on self-care agency and social support scales, respectively.

**Table 2:** Mean scores and correlation coefficient of HBP-SCP, ASAS-R, and MSPSS (N = 309)

Variables	Mean (SD)	1	2	3
1. Hypertension self-care profile (HBP-SCP)	37.7 (8.2)	1		
2. Appraisal of self-care agency (ASAS-R)	27.8 (7.0)	- 0.33*	1	
3. Multidimensional scale of social support (MSPSS)	40.2 (12.4)	0.35*	- 0.30*	1

*2-tailed *p*-value = 0.01

**Fig. 1:**
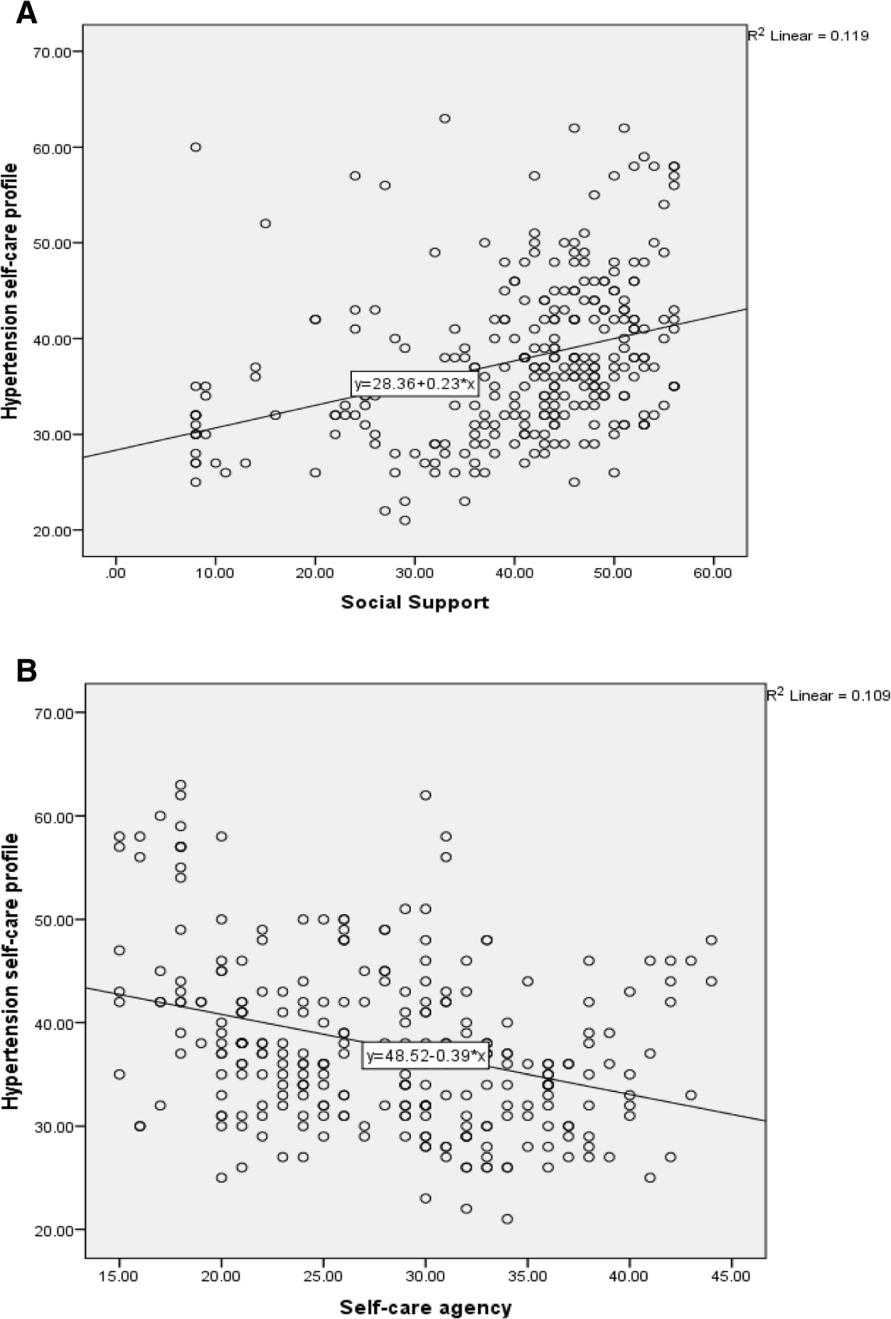
Correlation between hypertension self-care profile with social support (Pane **a**) and self-care agency (Panel **b**)

Figure 1 presents the correlation coefficient between hypertension self-care practice, self-care agency, and social support. Social support was positively correlated with hypertension self-care practice (R^2^ = 0.119, *p* = 0.01) while self-care agency was negatively correlated (R^2^ = 0.109, *p* = 0.01).

### Factors associated with hypertension self-care practice

In the simple logistic regression analysis, educational status of the participants, source of self-care information, income, gender, age, marital status, occupation, self-care agency, and social support had statistically significant association with hypertension self-care practice. However, in multiple logistic regression analysis only marital status, education, source of self-care information, place for exercise, social support, and self-care agency had statistically significant association with hypertension self-care practice (Table 3). Divorced participants were about 88% less likely to have good self- care practice compared to those who were single (AOR = 0.115, 95% CI = 0.026, 0.508, *p*-value < 0.01). Participants who had traditional clergy-based teaching were 2.2 times more likely to have good self-care practice compared to those who were unable to read and write (AOR = 2.209, 95% CI = 1.064, 4.584, *p*-value < 0.05). Compared to participant who obtained self-care information from books those who had no access to sources of information were about 92% less likely to have good self-care practice (AOR = 0.084, 95% CI = 0.022, 0.322, *p*-value < 0.01). Participant who had convenient place for exercise were about 3 times more likely to have good self-care practice compared to those who had not (AOR = 2.968, 95% CI = 1.826, 4.825, *p*-value < 0.01). Compared to participants with poor social support those who had good social support were 2.2 times more likely to have good self-care practice (AOR = 2.204, 95% CI = 1.272, 3.821, *p*-value < 0.01). Finally, participants who possessed good self-care agency were about 2 times more likely to have good self-care practice compare to those who had poor self-care agency (AOR = 1.222, 2.956, *p*-value < 0.05).

**Table 3:** Factors associated with hypertension self-care practice (*N* = 309)

Variable		Self-care practice		Crude Odds Ratio (COR), 95% CI	Adjusted Odds Ratio (AOR),	
		Good (n)	Poor (n)		95% CI	*p*-value
Gender	Male	80	64	1.0	1.0	
	Female	73	92	0.64 (0.40, 0.99)	0.58 (0.37, 0.92)	0.058
Age	<40 years	18	8	1.0	1.0	
	40–60 years	74	78	0.42 (0.17, 1.02)	0.32 (0.12, 0.83)	0.058
	>60 years	60	70	0.381 (0.16, 0.94)	0.33 (0.13, 0.88)	0.06
Marital status	Single	16	4	1.0	1.0	
	Married	108	106	0.26 (0.08, 0.79)	0.32 (0.09, 1.07)	0.17
	Divorced	8	20	0.1 (0.03, 0.39)	0.12 (0.03, 0.51)***	0.001
	Widowed	21	26	0.20 (0.06, 0.69)	0.27 (0.07, 1.06)	0.011
Educational status	Unable to read and write	33	67	1.0	1.0	
	Traditional clergy-based teaching	29	30	1.96 (1.02, 3.79)	2.21 (1.06, 4.58)**	0.036
	Primary school	33	36	1.86 (0.99, 3.49)	1.86 (0.92, 3.89)	0.046
	Secondary school	24	11	4.43 (1.94, 10.12)	2.52 (0.92, 6.91)	0.026
	College/university	34	12	5.75 (2.64, 12.53)	1.93 (0.71, 5.24)	0.013
Source of information	Books	24	4	1.0	1.0	
	News	0	1	0.00 [0.00]	0.0 (0, 0.00)	1.00
	Health education	103	83	0.21 (0.07, 0.62)	0.31 (0.09,1.05)	0.005
	No information	26	68	0.06 (0.02, 0.20)	0.08 (0.02, 0.32)***	0.000
Place for exercise	No	66	103	1.0	1.0	
	Yes	87	53	2.56 (1.62, 4.06)	2.97 (1.83, 4.83)***	0.000
Social support	Good social support	123	94	2.70 (1.62, 4.51)	2.20 (1.27, 3.82)***	0.005
	Poor social support	30	62	1.0	1.0	
Self-care agency	Good Self-care agency	65	98	2.29 (1.45, 3.16)	1.82 (1.12,2.96)**	0.015
	Poor Self-care agency	88	58	1.0	1.0	

**p-value < 0.05; ***p-value < 0.01

## Discussion

In this study, we explored the level of self-care practice and associated factors among hypertensive patients in public health facilities of Dessie town. Of the total participants, 51% had poor hypertension self-care practice. Poor self-care practice can be associated with increased prevalence of uncontrolled hypertension. Previous study reported a high prevalence of uncontrolled hypertension in Ethiopia [33]. Poor self-care practice may also contribute to the high risk of developing cardiovascular and renal complications among hypertensive patients.

Our study identified that marital status, education, source of self-care information, place for exercise, social support, and self-care agency have strong association with hypertension self-care practice. Compared to single hypertension patients those who were divorced engaged less in self-care practice. Previous studies in other disease conditions have also shown the influence of divorce on self-care behaviors [54]. This may be linked to the detrimental effect of divorce on mental and physical health status a person [55, 56] that in turn affects the ability to perform self-care activities [57]. Thus, divorced hypertension patients need to be given due considerations when designing and implementing self-care interventions.

This study revealed that hypertensive patients who had traditional clergy-based teaching were better off in self-care practices compared to those who cannot read and write. This corroborates with the findings of studies from other settings that identified lack of education and poor health literacy as risk factors for non-adherence to the recommended hypertension self-care practices. [35, 37]. Possessing the at least traditional clergy-based education could enable the patient to understand and follow the recommended self-care practices. This implies the need to design an educational intervention convenient for those who cannot read and write.

The presence of a convenient place for exercise is also an important predictor of hypertension self-care practice in this study. This finding corroborates with the established evidence that safe, walkable, and aesthetically pleasant physical environment positively influence individuals participation in physical activity [58–60]. Thus, facilitating physical activity among hypertensive patients require efforts in environmental planning and policy change. It may also requires creating community awareness about the importance of modifying the aesthetic nature of the local environment such as footpaths and trails, increasing accessibility of places to walk to, and reducing the level of road traffics for health.

Both social support and self-care agency were also good predictors of hypertension self-care practice in this study. Though limited, previous studies have also shown that higher socials support [41, 61] and self-care agency [62] are positively associated with hypertension self-care practices. This implies the need to develop strategies to improve social support and self-care agency in order to enhance hypertension self-care practice. Prospective studies are also needed to determine the effects of social support and self-care agency on hypertension self-care practice.

Our study shows that women hypertensive patient and those above 40 years of age were less likely to engage in hypertension self-care practice, using the 95% CI. This is contrary to the findings of a study conducted among African American, in which older adults and women more likely engaged in hypertension self-care [39]. The discrepancy may be linked to the difference in exposure to self-care information and the tools used the measure self-care.

### Limitations

This study has numerous limitations. Firstly, the use of cross-sectional design does not allow inferring causality. Prospective and experimental studies are warranted. Secondly, self-report measures were used for data collection, thus subject to recall bias that may affect the precision of measurement. Thirdly, we were not able to measure blood pressure due to lack of resources to purchase standardized measurement apparatus. Fourthly, even though our sampling approach was robust, we are not sure that the recruited participants were the same as those who were not recruited. This might have been source of selection bias. Finally, some potential influencing factors such as self-esteem, perceived health state, and health care experience were not measured in this study.

## Conclusion

Most of the hypertensive patients in this study reported poor self-care practices. Living in divorce status, the inability to read and write, lack of source of self-care information, lack of place for exercise, poor social support, and poor self-care agency may be the predisposing factors for this poor hypertension self-care practice. These findings suggest that patient’s marital status, the ability to read and write, and the presence of convenient environment for physical activity should be considered when planning hypertension self-care education. Instituting strategies to improve social support and self-care agency combined with prospective studies to determine their effective on hypertension self-care practices is also implied.

## Abbreviations

ASAS-R: Appraisal of Self-Care Agency-Revised
CI: Confidence interval
DALYS: Global disability-adjusted life years
HB-SCP: Hypertension Self-Care Profile
HPSCSM: Health-Promoting Self-Care System
MSPSS: Multidimensional Scale of Perceived Social Support
SPSS: Statistical Package for Social Sciences
WHO: World Health Organization
